# BXL 628 ameliorates toxicity of lactated Ringer in HK-2 human renal proximal tubule cells in a hypovolemia mimicking model

**DOI:** 10.1186/cc13346

**Published:** 2014-03-17

**Authors:** YT Huang, CC Cheng, TC Lin, PC Lai

**Affiliations:** 1 Buddhist Tzu Chi General Hospital, Hualien, Taiwan

## Introduction

Lactated Ringer (L/R) for resuscitation of hemorrhagic shock is suggested by the ATLS program. However, prior studies showed that resuscitation with L/R was associated with more kidney damage in rats with severe hemorrhagic shock. The direct effects of L/R on human renal tubule cells have not been reported.

## Methods

Human proximal renal tubular cell line HK-2 was used. Viability was examined by MTT assay. An additional equal volume of phosphate- buffered saline (PBS) served as control. Addition of 200 nM dipyridyl and 1 mM H_2_O_2 _served as conditions of hypoxia and oxidative stress, respectively. Patterns of cell death were observed by flow cytometry.

## Results

**Figure 1:**
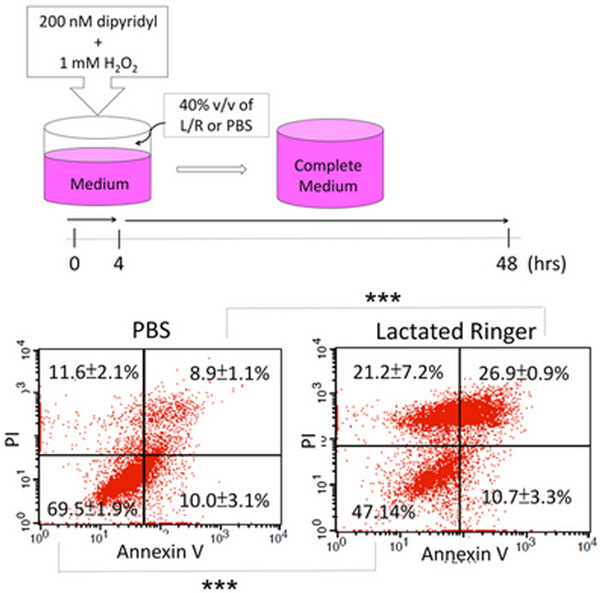
**Cell death of HK-2 cells after lactated Ringer administration in a hypovolemia mimic model**.

**Figure 2:**
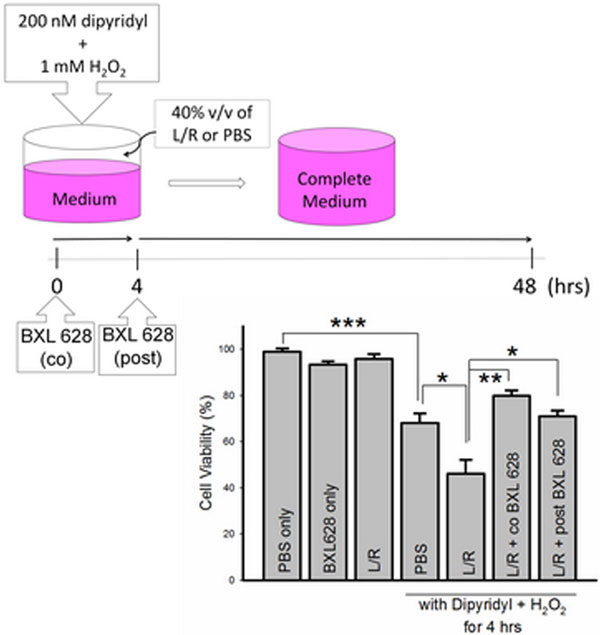
**Elocalcitol (BXL 628) ameliorates toxicity of lactated Ringer in HK-2 human renal tubule cells**.

To imitate early resuscitation in hypovolemic shock, medium was replaced with 40% v/v of L/R with dipyridyl plus H_2_O_2 _for 4 hours, followed by replacing with complete medium for 44 hours. In such conditions, L/R augmented cytotoxicity, and more annexin V (+) cells were observed. Co-treatment or post-treatment with BXL 628, a novel VDR agonist, reversed L/R-induced cytotoxicity. See Figures 1 and 2.

## Conclusion

BXL 628 rescued L/R-induced apoptosis in human renal proximal tubule cells in a hypovolemia mimicking model.

